# Relabeling the Medications We Call Antidepressants

**DOI:** 10.6064/2012/965908

**Published:** 2012-06-04

**Authors:** David Antonuccio, David Healy

**Affiliations:** ^1^Department of Psychiatry and Behavioral Sciences, University of Nevada School of Medicine, Reno, NV 89503, USA; ^2^Department of Psychology, Fielding Graduate University, Santa Barbara, CA 93105-3538, USA; ^3^Bangor University, Gwynedd LL57 2PW, UK

## Abstract

This paper raises the question about whether the data on the medications we call antidepressants justify the label of antidepressant. The authors argue that a true antidepressant should be clearly superior to placebo, should offer a risk/benefit balance that exceeds that of alternative treatments, should not increase suicidality, should not increase anxiety and agitation, should not interfere with sexual functioning, and should not increase depression chronicity. Unfortunately, these medications appear to fall short on all of these dimensions. Many of the “side effects” of these medications have larger effect sizes than the antidepressant effect size. To call these medications antidepressants may make sense from a marketing standpoint but may be misleading from a scientific perspective. Consumers deserve a label that more accurately reflects the data on the largest effects and helps them understand the range of effects from these medications. In other words, it may make just as much sense to call these medications antiaphrodisiacs as antidepressants because the negative effects on libido and sexual functioning are so common. It can be argued that a misleading label may interfere with our commitment to informed consent. Therefore, it may be time to stop calling these medications antidepressants.

## 1. Introduction

The medications we call antidepressants are incredibly popular. According to pharmaceutical consulting firm IMS Health, worldwide revenue estimates for antidepressants topped $20 billion in 2008, with almost $12 billion annually in the USA alone [1]. Estimates are that about 1 in 8 adult Americans had taken an antidepressant in the prior 10 years [2]. Of those taking antidepressants, about 60% indicate they have taken them for more than 3 months; 46% have taken them for more than a year. The CDC [3] found that antidepressant use has increased almost 400% in the USA since 1988, making antidepressants the most frequently used medications by people aged 18–44. The CDC study [3] also found that 11% of Americans aged 12 and older took antidepressants during the 2005–2008 study period. Less than 1/3 of Americans taking one antidepressant and less than 1/2 of those taking multiple antidepressants have seen a mental health professional in the prior year. Almost 25% of American women aged 40 to 59 are taking antidepressants. According to IMS Health [1], in 2010 more than 250 million prescriptions for antidepressants were filled in the USA, making them the number 2 most popular class of drug, just behind lipid regulators. One reason for their popularity is that primary care doctors are prescribing more than 73% of all antidepressants, most of the time without noting a psychiatric diagnosis [4]. In other words, these medications are being prescribed for the symptoms of depression, not just the diagnosis of depression.

## 2. An Antidepressant Should Be Clearly Superior to Placebo

These medications were originally developed because of a possible psychotropic drug effect that might be beneficial to patients diagnosed with depression [5]. To be labeled an antidepressant, a medication should be consistently and clearly superior to a sugar pill. Several meta-analyses have been conducted examining randomized controlled trials to determine whether this is so.

Kirsch et al. [6] used the Freedom of Information Act (FOIA) to access 38 randomized controlled trials (RCTs) involving 6944 patients from the USA Food and Drug Administration (FDA) database. These were all the RCTs used in the initial approval of the six most popular antidepressants. These included all of the available studies for fluoxetine, paroxetine, sertraline, venlafaxine, nefazodone, and citalopram, published or not. The modal duration of treatment was 6 weeks. This analysis showed that placebo duplicated 82% of the antidepressant response. This means that the placebo patients did almost as well as the patients on active medication. The average difference between the active drug and the placebo was less than 2 points on the Hamilton Depression Rating Scale (HDRS) [7]. Only 43% of the trials favored the antidepressant over placebo.

Kirsch et al. [8] conducted a subsequent meta-analysis of antidepressants that included all studies submitted to the FDA, whether published or not, for fluoxetine, nefazodone, venlafaxine, and paroxetine. The meta-analysis was limited to these 4 medications because the researchers decided to include studies only on those medications for which mean change scores were available on all trials. This analysis examined depression severity in relation to response. The results showed that the active drug only had clinically significant benefit (using the threshold for a clinically significant difference of ≥3 on the HDRS established by the National Institute for Clinical Excellence (NICE)) for those patients with an initial HDRS score greater than 28. In other words, Kirsch and colleagues conclude that the antidepressants had a clinically meaningful impact only on depressed patients in the very severe range. 

Fournier et al. [9] conducted a similar meta-analysis in which they analyzed 6 RCTs comparing a selective serotonin reuptake inhibitor (SSRI) and placebo. These researchers restricted their analysis to those studies that did not use a placebo washout (i.e., the common practice of offering all study participants a placebo for 2 weeks, and excluding placebo responders from the study). This was done to make sure that the studies were not biased against the placebo condition. They also only included studies for which they were able to get individual level data from the original researchers. This was done to ensure that no data were excluded in the analysis. Most meta-analyses use summary statistics generated from study publications rather than individual level data that can be independently analyzed. They were able to get individual level data on 718 patients. The analysis showed that antidepressants did not cause clinically meaningful benefits compared with placebo (also using the NICE threshold for a clinically significant difference of ≥3 on the HDRS) until patients had an initial intake HDRS score of 25. The authors note that this represents less than 30% of patients who seek treatment in clinical settings. In other words, similar to the Kirsch et al. [8] findings, Fournier et al. [9] concluded that only patients with very severe depression seemed to experience meaningful benefit from the antidepressant compared with a sugar pill. 

Through the Freedom of Information Act, Turner and his colleagues [10] reviewed 74 trials of 12 antidepressants submitted to and approved by the FDA. They found that selective publication of results of antidepressant drug trials has resulted in biased conclusions about the effectiveness of antidepressant drugs. Of the 74 FDA-registered studies in the report, 38 (51%) were found to have positive results, all but 1 of which were published. There were 36 studies the FDA found to have negative results. Of these, 3 were published with negative results (8%), 22 were not published, and 11 (33%) were published as if the results had been positive—directly conflicting with the FDA conclusions concerning outcome. Thus, while 94% of publications on antidepressants report success, the actual rate is 51%. Turner et al. [10] also found that the published literature inflated effect sizes (compared with effect sizes that include all of the FDA data) from 11% to 69%, averaging 32%. The authors point out that such selective and inflated reporting is misleading health care professionals and patients about the effectiveness of these medications. Of all the human subjects who participated in the studies included in this meta-analysis, 3449 never had their data published. An additional 1843 human subjects had their data positively spun in conflict with the FDA analysis. This was often accomplished by emphasizing positive secondary outcomes or by omitting nonsignificant prespecified primary outcomes altogether. Not publishing data or spinning data contrary to actual results would seem to be a violation of the IRB contract with human subjects [11]. Given that the FDA requires only 2 positive studies for approval of a psychotropic medication, many of the approved antidepressant medications have more negative studies than positive ones, for example bupropion, citalopram, paroxetine, and sertraline [10]. 

Based on the foregoing analyses, it would seem that medications called antidepressants are not more effective than a sugar pill at relieving depression for the vast majority of patients who take them. To be clear, it appears that many depressed patients improve on antidepressants, but this is also true of those who take placebos. However, the real-world outcomes with antidepressants may actually be much worse than those in the placebo-controlled trials. The STAR*D [12] study, a large (*N* = 4.041 depressed patients) and well-funded (35 million dollars from NIMH) study, was designed to mimic the real word. Depressed patients who were not helped by their first antidepressant received up to three additional trials with pharmacologically distinct treatments. This was designed to maximize the likelihood of obtaining and maintaining remission of depression via antidepressant medication. The medications used alone or in combination were Celexa, Zoloft, Effexor, Wellbutrin, Remeron, and Pamelor. Surprisingly, the data show that after a year of continuation treatment following remission, of the 4,041 patients who entered the study, only 108 (3%) had a sustained remission—all the other patients either dropped out or relapsed [12]. These actual results are in stark contrast to the STAR*D publicized cumulative remission rate of 67% theoretically attainable after four acute treatment steps. 

The results of STAR*D suggest that while the placebo-controlled studies provide evidence of an effect (i.e., a signal that the medication might be effective), this may not translate into effectiveness in the real world [13]. Furthermore, there is evidence that SSRIs are not effective with melancholic depression [14]. These patients tend not to qualify for the trials in the first place (i.e., most suicidal patients are excluded by design). 

## 3. Antidepressants Should Offer a Risk/Benefit Balance That Exceeds That of Alternatives

For medications to be considered true antidepressants, they should clearly offer benefit that exceeds the risks and side effects. To determine this, it is important to examine studies that compare these medications to credible nondrug interventions. Several studies allow such a comparison. Dimidjian et al. [15] randomly assigned 241 patients with major depression to paroxetine, cognitive therapy, behavioural activation, or placebo. The active treatments lasted 16 weeks while, for ethical reasons, the placebo treatment was limited to 8 weeks. All of the active treatments were superior to placebo after 8 weeks with behavioral activation having the best outcome in terms of response and remission at 16 weeks, followed by cognitive therapy, followed by paroxetine. After the acute phase of treatment, patients in the paroxetine condition were randomly assigned to continued placebo or continued paroxetine for one year. The cognitive therapy and behavioral activation conditions had treatment discontinued. The continued paroxetine condition and the discontinued psychotherapy conditions had similar survival rates (i.e., 55% to 65% of remitted patients remained remitted), while the newly assigned placebo patients deteriorated more rapidly (i.e., 40% remained in remission). After 1 year of follow-up, the patients who were continued on paroxetine had their medication stopped. They relapsed at a very high rate (i.e., only 15% sustained remission) while the discontinued behavioral activation and cognitive therapy patients did much better (i.e., about 50% of the remitted patients sustained remission). These authors concluded that the psychotherapy conditions had a clear cost advantage over medication at about 9 months after treatment initiation because of a more enduring benefit for the psychotherapies and the ability to discontinue treatment for most patients. 

Several other well-controlled trials have shown that psychotherapeutic interventions offer more enduring benefit than medications called antidepressants, even for severe depression [eg., [16–20]]. Even for patients who have “responded” to these medications, almost half indicate that they would not take them again due to unwanted psychological side effects such as narrowing of affect, not feeling like oneself, loss of creativity, and an inability to cry [21]. Physical side effects most often reported included sexual dysfunction, dry mouth, jitteriness, nausea, headaches, sweating, dizziness, lethargy, and inability to sleep [21].

## 4. An Antidepressant Should Not Increase Suicidality

The FDA analysis of the SSRI and SNRI database of medications called antidepressants trials in depressed youth (24 trials involving a total of 4,400 patients) found suicidal ideation and behavior in approximately 4% of those patients randomly assigned to the antidepressant compared with 2% of those randomly assigned to placebo [22]. While the risk of increased suicidality appears to be relatively low (i.e., two extra suicidal patients for every 100 treated with an antidepressant compared with a placebo) and no patients actually completed suicide in the FDA database of controlled trials, the stakes are clearly high. Another analysis using different statistical methods found 3% suicidality in the medication conditions versus 2% suicidality in the placebo conditions [23]. Unfortunately, data concerning potential risk are limited because randomized trials involving antidepressants have typically excluded suicidal patients. The acceptability of the risk/benefit profile with fluoxetine, the only antidepressant to show evidence of some benefit in depressed youth and the only antidepressant approved by the FDA for use with depressed children and adolescents, involves value judgments about the cost of harm-related and psychiatric-related adverse events. A legitimate question is ‘‘How many children should benefit from an antidepressant to justify one extra child harmed by an antidepressant?”

Whittington et al. [24] reviewed all of the available data (published and unpublished) from controlled trials of SSRIs in depressed youth. This meta-analysis concluded that the risk benefit profile (number needed to treat to benefit one extra patient (NNTB), versus number needed to treat to cause a serious adverse harm event in one extra patient (NNTH)) was favorable for fluoxetine but was unfavorable for paroxetine, sertraline, citalopram, and venlafaxine [25]. This analysis from Whittington et al. [24] did not include the Treatment for Adolescents with Depression Study (TADS), which did not show an advantage of fluoxetine alone compared with placebo. 

## 5. Antidepressants Should Not Increase Anxiety and Agitation

The Treatment of Adolescent Depression Study [26], conducted more recently than the studies included in the Whittington et al. [24] review, offers some of the most complete data relevant to the short-term relative risks of treating patients with psychotherapy alone, medication alone, the combination, or a placebo. Despite the fact that suicidality decreased across all four arms of this study, the fluoxetine condition had a significantly higher rate of harm-related adverse events (such as suicidal ideation), physiological side effects (diarrhea, insomnia, and sedation), and psychiatric adverse events (irritability, mania, and fatigue) compared with placebo or CBT alone. Using the global response measure from the TADS study, the NNTB is about three in the combined condition, five for fluoxetine alone, and 12 for CBT alone, all compared to placebo. In terms of harm-related adverse events, the NNTH is approximately 20 in the fluoxetine-containing conditions in comparison to nonmedication conditions. When considering psychiatric-related adverse events, the NNTH is approximately 10 in the fluoxetine alone condition compared with placebo and only about five compared with CBT alone. In other words, when considering psychiatric adverse events, a practitioner would only have to treat 5 patients with fluoxetine to harm one extra patient compared with treating those same 5 patients with CBT. Adding together the risk for psychiatric and physiological side effects and harm-related events reduces the NNTH for fluoxetine even further.

Follow-up to TADS found no significant differences in depression outcome in the three treatment groups at 36 weeks [27] or at 5 years [28]. However, the fluoxetine condition had significantly more suicidal events than CBT alone or the combination treatment at 36 weeks [27]. As concerning as this information may be, there are data to suggest that TADS [26, 27] underestimated the actual suicidality risk by prescribing antidepressants to some patients in the placebo or CBT conditions following the acute treatment phase [29]. When these newly prescribed patients had a suicidality event, it was apparently charged against their original nondrug assignment in the data analysis (uncovered by Goran Hogberg, see [30]) rather than the medication (see figure 1 in [29]). Therefore, those on medication in TADS may have been more than 4 times as likely to have a suicidality event compared with those who were not, rather than about twice as likely as originally thought. 

Psychiatric adverse events are not a trivial concern. Preda et al. [31] found that more than 8% of patients admitted to the Yale psychiatric facility were admitted for antidepressant-induced mania. Such adverse events can be frightening, costly, and extremely disruptive to a patient's life. 

## 6. An Antidepressant Should Not Interfere with Sexual Functioning

Sexual side effects caused by antidepressant medications appear to be a bigger problem than first thought in the original clinical trials. Premarket trials estimated that 2–16% of patients taking SSRIs and SNRIs experienced sexual dysfunction [32]. Montejo et al. [33] examined outpatients (610 women and 412 men) with previously normal sexual function who were being treated with antidepressants from April 1995 to February 2000. All patients were interviewed with the Psychotropic Sexual Dysfunction Questionnaire. Sexual dysfunction was reported by 62% of the men and 57% of the women. Women reported more severe symptoms. Dysfunctions included decreased libido, delayed orgasm, inability to have an orgasm, or decreased arousal. The SSRIs and venlafaxine resulted in the highest rates of dysfunction. Comparable rates of sexual dysfunction have been found in a more recent study [34]. There is even evidence that some patients may experience genital anesthesia or pleasureless orgasm, a problem that for some patients may persist even after the medication is discontinued [32]. 

## 7. Antidepressants Should Not Increase Depression Chronicity

Through a mechanism known as “oppositional tolerance” [35, 36], it has been suggested that antidepressant medications may actually cause persistence of depression symptoms in some patients. This phenomenon has been referred to as “tardive dysphoria” [37]. Some intriguing clues about the possibility of this phenomenon may have appeared in one of the early landmark comparative studies. For example, in the NIMH collaborative depression study, patients who had received imipramine (a tricyclic medication) were more likely to seek treatment during the follow-up period, had a higher probability of relapse, and had fewer weeks of minimal or no symptoms compared with those who had taken placebo [38]. In a recent analysis to determine the safety of the placebo condition in the TADS study, Kennard et al. [39] actually found that participants initially assigned to placebo had a lower utilization of crisis intervention during follow-up than those initially assigned to the active drug conditions. 

The SSRIs were developed to act on the serotonin system by interfering with serotonin reuptake. However, the brain quickly (as soon as 2 days in animal studies) compensates for this increase in serotonin through the process of downregulation or reduction in the number of serotonin receptors [40, 41]. The permanence of these changes and the potential long-term consequences are not clear. Fava [42] speculated almost 20 years ago that the receptor changes, similar to those found in tardive dyskinesia, may in some cases be irreversible, and may increase the biological vulnerability to depression in some patients following drug withdrawal, especially after long-term use. Baldessarini [43] has suggested that since some studies show a shorter time to relapse after drug discontinuation than would be expected from pretreatment history and the rate of drug removal predicts the time to the first recurrent episode, the combination of long-term drug treatment followed by withdrawal may be a causal factor in depression recurrence. He goes on to raise the possibility that it may take months to reestablish a predrug level of neurophysiological and neuropsychological homeostasis. Further research is needed to evaluate this possible risk.

## 8. Conclusions

On all of the identified dimensions for what a medication should accomplish to be called an antidepressant, current medications we call antidepressants seem to fall short. They are not clearly superior to placebo for the vast majority of patients for whom they are prescribed. The risks appear to outweigh the benefits for many patients, risks that are serious enough to warrant black box warnings about increased suicidality for patients under the age of 25 issued by the FDA and other regulatory bodies. There is now worldwide consensus that these medications increase the risk of suicidality. They may even increase the chronicity of depression in some patients. Anxiety, agitation, gastrointestinal problems, and sexual dysfunction are the most common side effects. 

If we do not call these medications antidepressants, what are some alternative labels that may better fit the existing data? The effect sizes for many of the “side effects” are larger than the antidepressant effect sizes. Using labels like antiaphrodisiac medications, agitation enhancers, insomnia inducers, suicidality inducers, mania stimulators, or gas busters obviously would not offer the same marketing appeal. Though tongue in cheek, we consider these possible labels to be more accurate than the commonly used label of “antidepressant.” It could be argued that the outcomes with the largest effect sizes should be offered as the primary label for a medication. Though the data reviewed in this paper appear not to adequately support the label of antidepressant, as long as these medications continue to be called antidepressants, prescribers will feel a moral obligation to offer them to their patients who are suffering from depression. Of course, the drug industry does not have an incentive to change the label. However, we feel patients ought to be informed of these possible alternative labels because they may apply equally well if not better. The main point is that calling these medications antidepressants is a marketing decision that does not appear to be consistent with the scientific data. 
